# Prevalence of suicide risk among a national sample of individuals referred from a primary care subpopulation, 2017–2020

**DOI:** 10.1093/haschl/qxad029

**Published:** 2023-07-21

**Authors:** Virna Little, Ohshue S Gatanaga, Spencer Hutchins, Christian T Gloria

**Affiliations:** Concert Health, San Diego, CA 92101, United States; Concert Health, San Diego, CA 92101, United States; Department of Sociomedical Sciences, Columbia Mailman School of Public Health, New York, NY 10032, United States; Concert Health, San Diego, CA 92101, United States; Department of Sociomedical Sciences, Columbia Mailman School of Public Health, New York, NY 10032, United States

**Keywords:** suicide risk, screening, suicide prevalence, referrals, primary care

## Abstract

Over the past decade, the age-adjusted suicide rate has increased by 35.2% in the United States. In primary care, practitioners often interact with patients at risk of dying by suicide, yet little is known about the prevalence of suicide risk in primary care populations. Patient data from 2017–2020, consisting of a national sample of patients referred from primary care and enrolled in collaborative care behavioral health services (n = 37 666), were analyzed. Controlling for demographic characteristics, logistic models were used to compare suicide risk prevalence by behavioral health diagnosis. An estimated 9.96% (95% confidence interval [CI]: 9.65–10.27)—or approximately 3751 individuals—of the total sample screened positively for suicide risk. Compared with individuals diagnosed with generalized anxiety disorder, individuals diagnosed with bipolar disorder had 8.21 times the odds (95% CI: 6.66–10.10) of screening for suicide risk. Practitioners and health care systems may benefit from adding suicide risk screeners as a standard practice for referred patients, which may lead to further development of clinical pathways and provider training. The high rate of suicide risk across the sample suggests that more research is needed to understand suicide risk prevalence across primary care and collaborative care populations.

## Introduction

In the United States, suicide prevalence has risen across virtually all ages and populations and is among the top leading causes of death for individuals ages 10–64 years.^1^ The 2020 National Survey on Drug Use and Health found that over 12 million adults seriously thought about suicide, over 3 million made a plan for suicide, and over 1 million attempted suicide in the past 12 months.^2^ In primary care settings, suicide has increasingly become a concern as primary care providers (PCPs) commonly interact with individuals at risk of suicide.^3^ A growing number of primary care clinicians report addressing mental health concerns for US adults. Moreover, an estimated 45% of individuals who died by suicide had contact with PCPs within 1 month of their death, whereas nearly 75% of all individuals had contact with PCPs within 1 year of their death.^4^ Such opportunities to identify and treat patients at risk of suicide have led to numerous recommendations from researchers and health care organizations to implement partial or universal suicide screenings in primary care.^5–7^

Despite these recommendations, the US Preventive Services Task Force, while recommending universal depression screening in adults, concluded in 2022 that there is insufficient evidence for or against screening for suicide risk at the population level.^8^ In fact, some stakeholders argue that a lack of fidelity to suicide screening tools due to improperly trained or time-strapped PCPs will lead to false identification of individuals at risk of suicide, costing the health care system greatly through increased, unnecessary behavioral health care utilization.^9^ Regardless, inconsistent messaging around suicide management has contributed to a disjointed implementation of suicide screening across primary care and behavioral health providers more broadly. As such, there exists limited knowledge around the prevalence of suicide risk in primary care populations and associated behavioral health providers at the population level—this is particularly concerning as upwards of three-quarters of American adults visit their PCP annually.^10^ Of the existing population health data on suicide risk across the general public, the 2020 National Survey on Drug Use and Health estimates that approximately 4.8% of American adults reported having suicidal thoughts in the past 12 months.^2^ In 1 exploratory study analyzing youth ages 11 to 20 years in urban primary care systems, Gardner et al.^11^ found that 14% of their sample reported suicidal thoughts in the previous month.

Extant literature has also demonstrated the linkage between behavioral health conditions and suicide risk. Individuals diagnosed with schizophrenia, bipolar disorder, major depressive disorder, and other serious mental illnesses are at higher risk of dying by suicide.^12^ While the overarching literature notes the varying prevalence of these conditions in primary care, less is known about the relative risk between different behavioral health conditions among subgroups of primary care populations. On one hand, primary care physicians are well positioned to identify and address the needs of individuals with serious mental illnesses.^13^ On the other hand, given the resource-limited nature of primary care, identifying the patients who require the most attention among those with behavioral health diagnoses will inform PCPs of patients who may benefit most from monitoring and follow-up. This may be particularly important for behavioral health providers embedded in integrated-care models, who are at the forefront of practically managing suicidal crises in primary care settings.^14^

Further research at the population level is needed to grasp the true extent to which individuals are at risk of suicide so as to inform medical practice at the individual level and future policy decision making around suicide screening in primary care. As such, this study aims to estimate the prevalence of suicide risk among a national sample of individuals referred to collaborative care behavioral health services from primary care. Collaborative care is an evidence-based model supported by over 80 randomized controlled trials that identifies and treats individuals in primary care settings, typically those with mild to moderate anxiety and depression.^15^ While a subpopulation of the entire primary care population, an estimate of suicide risk prevalence among a national sample of individuals enrolled in collaborative care services provides implications for suicide screenings and interventions for these individuals referred from primary care. This study will also compare the prevalence of suicide risk across primary behavioral health diagnoses to better understand the subpopulations that may be most at risk of suicide, and therefore require more follow-up from PCPs and collaborative care clinicians. Current research on suicide risk among patients enrolled in collaborative care services has focused on suicide risk among individuals with depression and has yet to highlight suicide risk among individuals with other behavioral health diagnoses.^16^

## Data and methods

This study includes a total of 37 666 individuals who were referred for behavioral health treatment by PCPs across a population of 2377 providers, 676 practices including 4 large rural health centers and approximately 10 federally qualified health centers, and 13 states. The data collected were in the provision of collaborative care, an evidence-based model to identify and treat patients with depression and anxiety, from a behavioral health medical group providing remote collaborative care across 13 states through health care providers. The average primary care provider panel size included in the study is approximately 1500 patients. The data consist of one of the largest datasets for analysis examining primary care patients referred to behavioral health services. This study has a longitudinal design based on secondary data, from April 21, 2017, to October 31, 2022, and stored in the medical group's comprehensive registry.

The inclusion criteria for participants were as follows: (1) all patients enrolled in and closed from collaborative care services from April 21, 2017, to October 31, 2022; (2) who received a primary diagnosis during treatment; and (3) were referred to collaborative care services by their health care providers.

### Measures

Information on age, geographic area, insurance type, and primary diagnosis was collected from participants at the time of enrollment. The primary study outcome was suicide risk, which was assessed by Master’s-level clinicians and psychiatric nurse practitioners or physicians utilizing a combination of the following validated tools: item 9 on the 9-item Patient Health Questionnaire (PHQ-9) and the Columbia Suicide Severity Rating Scale (C-SSRS).^17,18^ A positive score on item 9 of the PHQ-9 and C-SSRS would indicate a positive suicide risk for the participant.^19^ Item 9 on the PHQ-9 addresses suicidal thoughts and asks whether individuals had “thoughts you would be better off dead or of hurting yourself in some ways” in the past 2 weeks.^17^ A positive suicide risk on the C-SSRS was assessed by whether individuals answered questions 4, 5, and 6 on the C-SSRS as positive: “Have you had thoughts of killing yourself and had some intention of acting on them?”; “Have you started to work out the details of how to kill yourself? Do you intend to carry out this plan?”; “Have you ever done anything, started to do anything, or prepared to do anything to end your life?”^18^ Together, affirmative answers to these questions indicate that an individual has not only thoughts of suicide and plans to die by suicide but intent to die by suicide.

### Statistical analysis

First, the prevalence of suicide risk with respect to age, geographic location, insurance type, and primary diagnosis was analyzed. Geographic location included Arizona, California, Connecticut, Florida, Missouri, New York, and other states. Other states included Arkansas, Georgia, Illinois, Massachusetts, New Mexico, Texas, and Washington. These states were grouped together, as the prevalence of suicide risk was not sufficient for analysis when individually analyzed. Primary diagnoses included generalized anxiety disorder, adjustment disorder, bipolar disorder, major depressive disorder, posttraumatic stress disorder, unspecified anxiety disorder, and other disorders such as schizophrenia, postpartum depression, and acute stress reactions that did not meet the prevalence of suicide risk sufficient for analysis. We conducted bivariate analysis to compare the prevalence of suicide risk according to the aforementioned demographic characteristics. Rao-Scott chi-square analysis was used for these comparisons. A multivariate logistic regression model was then used to compare the prevalence of suicide risk among patients by age, geographic location, insurance type, and primary diagnosis. Adjusted odds ratios (aORs) were derived from the multiple logistic regression models to indicate associations between the covariates. Given the large sample size, the level of significance was set at alpha = 0.01. R version 4.2.2 was used for all analyses.^20^ This study was determined to be exempt by the Columbia University Irving Medical Center Institutional Review Board.

## Results

### Demographic characteristics

Table 1 shows the demographic characteristics of the study sample. Specifically, a majority of the sample were between the ages of 18 and 45 years, with 24.32% (n = 9159) aged 18–30 years and 27.42% (n = 10 329) aged 31–45 years old. A total of 35.03% (n = 13 194) of patients were enrolled in New York, followed by 20.26% (n = 7630) in Arizona, 10.02% (n = 3776) in Missouri, 9.41% (n = 3543) in North Carolina, 8.45% (n = 3184) in California, 4.67% (n = 1760) in Connecticut, and 12.16% (n = 4579) in other states. With regard to insurance status, approximately half (50.38%; n = 18 978) of patients were enrolled in commercial insurance, 29.84% (n = 11 239) were enrolled in Medicaid, 8.52% were enrolled in Medicare (n = 3209), 8.99% (n = 3385) were enrolled in Medicare Advantage, and 2.27% (n = 855) paid for services out-of-pocket.

**Table 1.** Sample characteristics of individuals referred to collaborative care through primary care (n = 37 666).

Full sample (n = 37 666)	Frequency (valid %)
Age	
<18 y	2633 (6.99%)
18–30 y	9159 (24.32%)
31–45 y	10 329 (27.42%)
46–55 y	4906 (13.03%)
56–64 y	4475 (11.88%)
≥65 y	6164 (17.36%)
State	
Arizona	7630 (20.26%)
California	3184 (8.45%)
Connecticut	1760 (4.67%)
Florida	3543 (9.41%)
Missouri	3776 (10.02%)
New York	13 194 (35.03%)
Other	4579 (12.16%)
Insurance	
Commercial	18 978 (50.38%)
Medicaid	11 239 (29.84%)
Medicare	3209 (8.52%)
Medicare Advantage	3385 (8.99%)
Out-of-pocket	855 (2.27%)
Primary diagnosis	
Generalized anxiety disorder	9212 (24.46%)
Adjustment disorder	1405 (3.63%)
Bipolar disorder	590 (1.57%)
Major depressive disorder	15 131 (40.17%)
Posttraumatic stress disorder	497 (1.32%)
Unspecified anxiety disorder	7650 (20.31%)
Other	3181 (8.45%)
Total	37 666 (100.00%)

Source: Authors’ analysis of data from the 2015–2022 electronic records from a behavioral health medical group that partners with organizations to deliver collaborative care.

### Correlates of suicide risk

**Figure 1.**
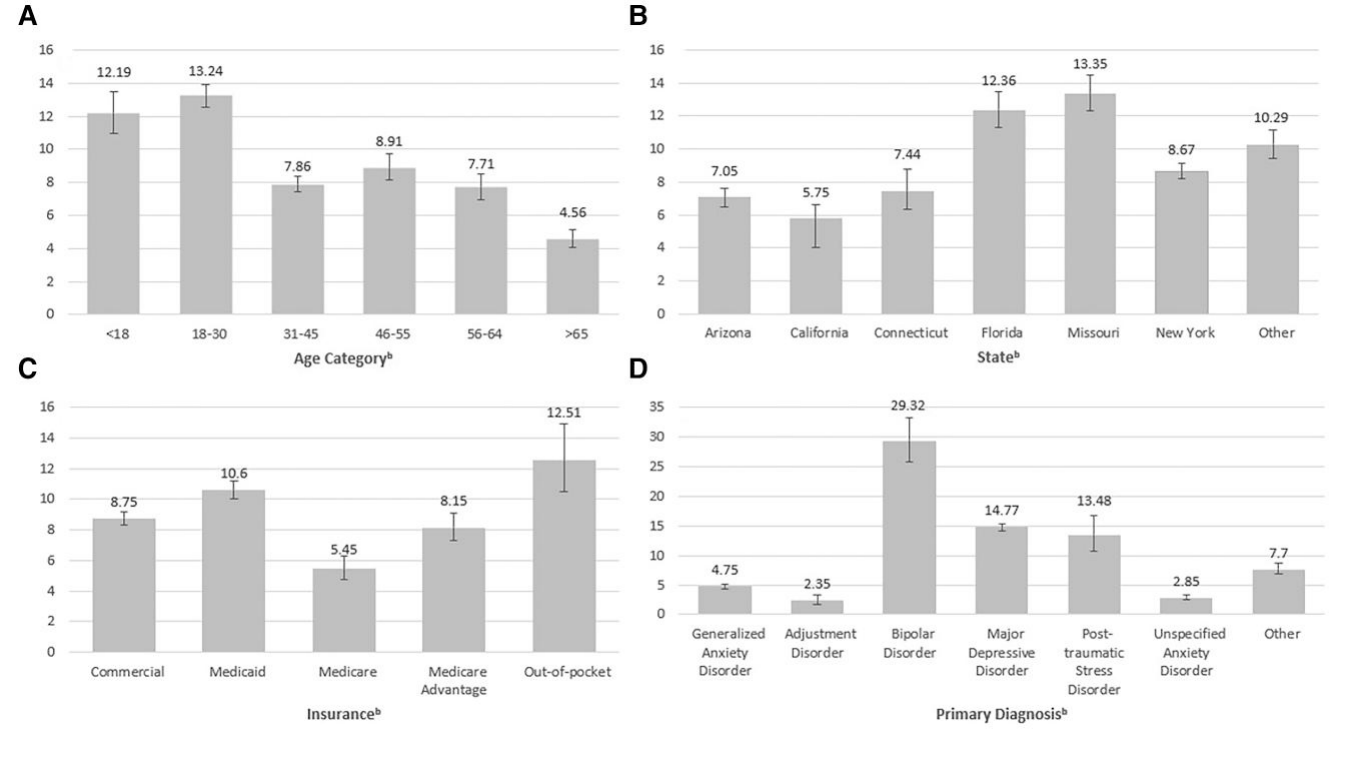
(A–D) Estimated prevalence of suicide risk and confidence intervals by sample characteristics among individuals referred to collaborative care through primary care (n = 37 666). Overall suicide risk prevalence was 9.96% (n = 3751). Bivariable tests were conducted. Bold type indicates statistical significance: ^a^*P* < .01; ^b^*P* < .001. Source: Authors’ analysis of data from the 2015–2022 electronic records from a behavioral health medical group that partners with organizations to deliver collaborative care.

Figure 1 depicts the prevalence of positive suicide risk by age, geographic location, insurance type, and primary diagnosis. Across the entire study sample, 9.96% (95% confidence interval [CI]: 9.64–10.27%) screened positive for suicide risk. Bivariate analysis indicated significant differences in positive suicide risk screening with regard to age (*P* < .001), state enrolled (*P* < .001), insurance type (*P* < .001), and primary diagnosis (*P* < .001). The multivariable model analyzing suicide risk by the demographic covariates also showed significant differences by age, state, insurance type, and primary diagnosis (Table 2). Compared with individuals younger than 18 years, the odds of a positive suicide screen was lower for all other age groups, with the lowest odds of positive suicide risk for individuals aged 65 years or older, who had 0.20 (95% CI: 0.16–0.25) times the odds of positive suicide risk. Among various states, compared with patients in Arizona, those in Florida (aOR = 1.61; 95% CI: 1.40–1.85) and Missouri (aOR = 1.57; 95% CI: 1.38–1.80) had higher odds of screening positively for suicide risk. For insurance type, compared with commercial insurance holders, all insurance types other than individuals enrolled in Medicaid had higher odds of suicide risk. In particular, individuals enrolled in Medicare Advantage had 1.62 (95% CI: 1.37–1.91) times the odds of positive suicide risk compared with the odds of positive suicide risk among individuals enrolled in commercial insurance. Analysis by primary diagnosis demonstrates that individuals diagnosed with adjustment disorder (aOR = 0.60, 95% CI: 0.41–0.85) and unspecified anxiety disorder (aOR = 0.62; 95% CI: 0.53–0.74) were at lower odds for suicide risk than individuals diagnosed with generalized anxiety disorder. On the other hand, individuals diagnosed with bipolar disorder (aOR = 8.21; 95% CI: 6.66–10.10), major depressive disorder (aOR = 3.79; 95% CI: 3.41–4.23), and posttraumatic stress disorder (aOR = 3.25; 95% CI: 2.44–4.27) had higher odds of positive suicide risk compared with individuals with generalized anxiety disorder.

**Table 2.** Correlates of suicide risk among individuals referred to collaborative care through primary care (n = 37 666).

Full sample (n = 37 666)	aOR	95% Confidence interval
**Age**		
<18 y	1.00	
18–30 y	0.95	(0.83, 1.09)
31–45 y	0.52	(0.46, 0.61)^b^
46–55 y	0.54	(0.46, 0.63)^b^
56–64 y	0.41	(0.35, 0.49)^b^
≥65 y	0.20	(0.16, 0.25)^b^
State		
Arizona	1.00	
California	0.84	(0.70, 1.01)
Connecticut	1.09	(0.89, 1.34)
Florida	1.61	(1.40, 1.85)^b^
Missouri	1.57	(1.38, 1.80)^b^
New York	1.10	(0.98, 1.23)
Other	1.34	(1.17, 1.53)^b^
Insurance		
Commercial	1.00	
Medicaid	1.11	(1.02, 1.21)
Medicare	1.43	(1.16, 1.74)^b^
Medicare Advantage	1.62	(1.37, 1.91)^b^
Out-of-pocket	1.39	(1.11, 1.72)^a^
Primary diagnosis		
Generalized anxiety disorder	1.00	
Adjustment disorder	0.60	(0.41, 0.85)^a^
Bipolar disorder	8.21	(6.66, 10.10)^b^
Major depressive disorder	3.79	(3.41, 4.23)^b^
Posttraumatic stress disorder	3.25	(2.44, 4.27)^b^
Unspecified anxiety disorder	0.62	(0.53, 0.74)^b^
Other	1.69	(1.43, 1.99)^b^

Abbreviation: aOR, adjusted odds ratio (controlling for all covariates presented in table).

Source: Authors’ analysis of data from the 2015–2022 electronic records from a behavioral health medical group that partners with organizations to deliver collaborative care. ^a^*P* < 0.01; ^b^*P* < 0.001.

## Discussion

The study presents results estimating the prevalence of suicide risk among a national sample of patients referred to collaborative care services from their PCPs. In particular, this study disaggregated the patients by primary behavioral health diagnosis and found key differences in suicide risk for individuals diagnosed with bipolar disorder, major depressive disorder, and posttraumatic stress disorder. These findings reinforce the existing literature that underscores especially high rates of suicide risk among individuals with bipolar or major depressive disorder.^12^ This study is one of the first to report the prevalence of suicide risk among a national sample of US individuals enrolled to behavioral health services, specifically collaborative care, from primary care settings.^21^ Across our entire sample of individuals referred from primary care to collaborative care services, the prevalence of suicide risk was estimated to be approximately 10% (n = 3751).

Overall, our findings provide policy implications on the use of depression and suicide screenings in patients referred to collaborative care from primary care settings that may result in a better understanding of suicide risk in primary care subpopulations. In particular, the findings suggesting higher rates of suicide risk among individuals with major depressive disorder highlight the significant role that depression screeners may play in identifying at-risk populations in primary care practices. While the US Preventive Services Task Force has recommended that universal depression screenings should be implemented in primary care practices, the national depression screening rate in 2015 was 1.4%, with an estimated 2.2% of primary care visits including depression screenings for adults.^8,22^ The low rates of depression screenings in primary care settings are concerning not only with regard to the lost opportunity in assessing and treating individuals with major depressive disorder but also for identifying individuals generally known to have a higher suicide risk. In this regard, PCPs should continue to—or start implementing—standard depression screeners as common practice.

In implementing depression screeners in primary care, the PHQ-9 may be of particular use as the gold-standard tool for measuring depressive symptomatology.^17^ In recent years, a growing body of literature has criticized the use of item 9 in the PHQ-9, which aims to evaluate passive thoughts of death or self-injury. Some claim that item 9 does not accurately assess suicidal ideation and should be removed, while others note that item 9 still has utility as an exploratory question around suicide and is remarkably reliable despite the item’s brevity.^23,24^ Insofar as there is no definitive consensus against the inclusion of item 9 in the PHQ-9, we believe that PCPs would benefit from utilizing the unabridged version of the PHQ-9 as an exploratory item around suicidal thoughts for individuals who may already carry depressive symptomology that increases their risk of dying by suicide. To this extent, the extant literature has noted the high reliability of utilizing a standard suicide screening in conjunction with the PHQ-9, given that a patient responds positively to item 9 of the PHQ-9.^19^ More research should be conducted to continually streamline this process of screening individuals for suicide risk.

Finally, this study offers an exploratory analysis around the prevalence of suicide risk among a primary care subpopulation. By gaining an understanding of the prevalence of patients at risk of suicide in patients referred from a primary care population, providers and organizations may be driven to implement systemic approaches to suicide care, as they have for other chronic illnesses such as cardiovascular disease and diabetes—especially in integrative care settings. As such, the implementation of suicide screenings in primary care settings—particularly among individuals delivering integrated behavioral health care and collaborative care providers—may embolden PCPs and behavioral health providers to gain insight into the size of their at-risk populations. Providers who begin to understand prevalence in the patients they referred for behavioral health treatment may increase the number of patients they refer for care, be more reflective of the population who need behavioral health care, and increasingly ask patients directly about suicide risk. Asking for suicide risk directly is an evidence-based suicide prevention practice that should be implemented within practices.^25^

Several limitations exist for the present study. The data are based on self-reported measures on the PHQ-9 and the C-SSRS and are therefore subject to recall and reporting bias. Additionally, the data do not include demographic variables such as gender, socioeconomic status, and race/ethnicity that may serve as effect modifiers or confounders for our suicide risk estimates. Our data are cross-sectional and are neither predictive of suicide risk in the future nor the past. Data provided by the behavioral medical group are in the provision of a predominantly remote collaborative care service. While collaborative care services have generally been designed to be implemented telephonically, the generalizability of our results to other in-person collaborative care providers may be limited.^15^ Last, the external validity of our results remains somewhat limited as the sample specifically includes only patients who were referred from primary care to collaborative care services. For example, the results indicate that 40.17% of the study sample were diagnosed with major depressive disorder, although data from the US Preventive Service Task Force suggest that the national prevalence of depression is estimated at 8% for persons aged 12 years and older.^26^ Our study sample likely oversamples individuals with mild to moderate depressive symptoms, and thus the results of the study may not be generalizable outside of the primary care subpopulations referred to collaborative care services.

In light of these limitations, our study provides an exploratory understanding of the prevalence of suicide risk in a national sample of a primary care subpopulation. The study hopes to add to the literature around implementation of continued depression screenings for identifying at-risk populations, highlight key differences in suicide risk by primary behavioral health diagnosis, further contextualize the debate around implementing suicide screenings in primary care subpopulations, and encourage the management of at-risk suicide populations like other chronic diseases. While the study implications and generalizability remain somewhat limited, they indicate the need for further research around the prevalence of suicide risk in other primary care subpopulations, the broader primary care population, and related risk factors. Overall, this study is a starting point for health care providers and organizations to continually reflect on the reality that suicide is an increasingly present, yet addressable, national crisis at the primary care level. Screening for suicide risk, and reflecting on individual and institutional practices around suicide screenings, is an opportunity for health care providers to directly address the suicide crisis in the United States. An understanding of the national prevalence of suicide risk among a primary care subpopulation may thus encourage providers to proactively streamline their suicide screening processes, especially among patients who may be more at risk of suicide.

## Supplementary Material

qxad029_Supplementary_Data
